# Combining of transcriptome and metabolome analyses for understanding the utilization and metabolic pathways of Xylo‐oligosaccharide in *Bifidobacterium adolescentis* ATCC 15703

**DOI:** 10.1002/fsn3.1194

**Published:** 2019-09-30

**Authors:** Jian Yang, Qilong Tang, Lei Xu, Zhijiang Li, Yongqiang Ma, Di Yao

**Affiliations:** ^1^ Department of Food and Engineering College of Food Engineering Harbin University of Commerce Harbin China; ^2^ Department of Food and Engineering College of Food Heilongjiang Bayi Agricultural University Daqing China

**Keywords:** *Bifidobacterium adolescentis*, metabolome, transcriptome, Xylo‐oligosaccharide

## Abstract

A combination of transcriptome and metabolome analyses was applied to understand the utilization and metabolism of Xylo‐oligosaccharide (XOS) in *Bifidobacterium adolescentis* 15703 as well as identifying the key regulatory‐related genes and metabolites. Samples of cultures grown on either XOS or xylose were collected. The transcript and metabolite profiles were obtained from high‐throughput RNA‐sequencing data analysis and UHPLC system. Compared with xylose, XOS highly promoted the growth of *B. adolescentis* 15703 and resulted in a growth yield about 1.5‐fold greater than xylose. The transcriptome analysis showed that XOS could enhance genes, including ABC transporters, galactosidase, xylosidase, glucosidase, and amylase, which were involved in transport and metabolism of carbohydrate compared with xylose. Furthermore, the expression profile of 16 candidate genes using qRT‐PCR has validated the accuracy of the RNA‐seq data. Also, the metabolomic analyses, particularly those related to metabolic biomarkers of fatty acids, amino acids, and sugars showed a similar trend of result and approved the advantages of XOS as growth medium for *B. adolescentis* 15703 compared with xylose. The expression and abundance of specific genes and metabolites highlighted the complex regulatory mechanisms involved in utilization of XOS by *B. adolescentis* 15703. These results are useful in the understanding of the metabolic pathway of XOS in *B. adolescentis* 15703 and contribute to the optimization of XOS probiotic effects as a food additive.

## 1 INTRODUCTION

*Bifidobacterium* is a genus of gram‐positive bacteria that colonize in the human gastrointestinal tract and provide health benefits. Several studies verified a wide range of positive effects for *Bifidobacterium*, including the protection against pathogenic bacteria, alleviation of allergic disease symptoms (Casaro et al., 2018), immune regulation, reduction in intestinal inflammations, and the potential of bifidobacteria to prevent and/or treat colorectal cancer (Le Leu, Hu, Brown, Woodman, & Young, 2010). Due to claimed health benefits, bifidobacteria has been incorporated into many functional foods (O'Callaghan & van Sinderen, 2016). Therefore, more health benefits are expected if the amount of bifidobacteria could be increased in the body.

Xylo‐oligosaccharides (XOSs) are hydrolysates of xylan and consist of a backbone of xylose, which are noncaloric and indigestible by humans. XOSs are believed to exert bifidogenic effects and are increasingly used as prebiotics. XOS may be beneficial in stimulating the intestinal *Bifidobacterium* without significant effect on *lactobacillus* (Li, Summanen, Komoriya, & Finegold, 2015; Falck et al., 2013). Also, it was found that XOS increases bifidobacteria, but not lactobacilli in human gut microbiota (Finegold et al., 2014). Due to potential bifidobacteria proliferation effects, XOSs have attracted increasing interest.

Carbohydrate metabolism may vary among bifidobacterial strains considerably (Pokusaeva, Fitzgerald, & Sinderen, 2011). *Bifidobacterium adolescentis* has the ability to utilize XOS efficiently (Amaretti et al., 2013). Bifidobacteria lack a number of key enzymes involved in the Embden–Meyerhof–Parnas (EMP) pathway; therefore, bifidobacteria metabolize carbohydrates through a metabolic pathway named the “bifid shunt,” which is centered on the key enzyme fructose‐6‐phosphoketolase (De Vries & Stouthamer, 1967). In a previous study, we have found that the growth rate of *B. adolescentis* was higher in the presence of XOS than xylose (unpublished). However, the underlying molecular regulation mechanisms of XOS metabolism are not fully understood. In XOS utilization process, xylose is not neatly consumed and remaining unfermented (Amaretti et al., 2013). Although it has been established that XOSs confer positive benefits to bifidobacteria, there is a lack of knowledge regarding the molecular mechanisms that explain the metabolic pathway of XOS in *B. adolescentis*. Meanwhile, a recent study performed on the genome sequences from 47 *Bifidobacterium* (sub) species found that 5.5% of the core bifidobacterial genomic coding sequences are associated with carbohydrate metabolism (Pokusaeva et al., 2011). Therefore, an in‐depth study on these functional genes has significance for understanding mechanisms of probiotic effects of *Bifidobacterium*. In this work, a combination of transcriptome and metabolome analyses was applied to elucidate the molecular mechanism for utilizing and metabolism of xylose and XOS in *B. adolescentis* 15703. Understanding of basic mechanisms may help in finding of novel ways to optimize the use of prebiotics and probiotics in the food industry.

## 2 MATERIALS AND METHODS

### 2.1 Materials

*Bifidobacterium adolescentis* ATCC 15703 was purchased from China General Microbiological Culture Collection Center. XOS extracted from corncob, 95% purity, DP of 2–7 and containing 22.76% xylobiose, 31.45% xylotriose, 20.37% xylotetraose, 10.89% xylopentaose, 4.68% xylohexaose, and 6.37% wood seven sugar was obtained from LongLive Biotechnology. All other chemicals were of analytical grade.

### 2.2 Bacterial cultivation and carbohydrates fermentation

*Bifidobacterium adolescentis* 15703 was resuscitated and precultivated twice using MRS broth. Cells were harvested and suspended as 2% inoculate into MRS medium containing xylose or XOS as well as a control medium without carbohydrate and incubated at 37°C under anaerobic conditions. Aliquots of cultures were drawn at regular intervals and cell growth was determined by measuring the optical density at 600 nm (Lei et al., 2018).

### 2.3 RNA extraction

Cells were harvested from triplicate cultures at the estimated early midexponential growth phase by centrifugation at 4,000 *g* for 10 min at 4°C for RNA isolation and purification. The samples were used for RNA extraction following the manufacturer's recommendations of QIAGEN 74524 kit. RNA concentration was determined with a Qubit RNA Assay Kit in a Qubit 2.0 fluorometer (Life Technologies). RNA purity and integrity were assessed by a Nanodrop spectrophotometer (IMPLEN).

### 2.4 Library construction and sequencing

After total RNA extraction, prokaryotic mRNA was enriched by removing rRNA using Ribo‐Zero™ Magnetic Kit (Epicentre). Then the short fragments were obtained from the enriched mRNA by fragmentation buffer and were reverse transcripted into cDNA. Under the action of DNA polymerase I, RNase H and dNTP, second‐strand cDNA was synthesized. Then, the cDNA fragments were purified, end repaired, poly (A) added, and ligated to Illumina sequencing adapters (Bellieny‐Rabelo et al., 2019). The ligation products size were chosen, amplified, and sequenced using Illumina HiSeq™ 2500.

### 2.5 Transcriptomic analysis

Raw reads were filtered to remove some adapters and low‐quality reads, and the remaining reads were mapped to a reference genome by TopHat2 (Kim et al., 2013). The reconstruction of transcripts was carried out with software Cufflinks (Trapnell et al., 2012), then the transcripts were merged from multiple groups into a finally comprehensive set of transcripts for further downstream differential expression analysis. Gene abundances were quantified by software RSEM (Li & Dewey, 2011). The gene expression level was normalized with FPKM method, and the edgeR package was used to identify DEGs across groups. In comparison as significant DEGs, FDR <0.01 and fold change (FC) ≥2 were used as screening criteria. We conducted gene expression differences between xylose and XOS treatments using the DEseq package. DEGs were then subjected to enrichment analysis of COG functions and KEGG pathways.

### 2.6 Confirmation of transcriptomic results by quantitative real‐time PCR

Total RNA was isolated as described above. Using a Revert Aid Premium Reverse Transcriptase, the cDNA synthesis was performed. qRT‐PCR primers are listed in Table 1 and each reaction (20 μl mixture) contained 2 μl cDNA, 10 μl 2 × sybrGreen qPCR Master Mix, 0.4 μl the forward and reverse primers and 7.6 μl water. All qRT‐PCR were performed in ABI Stepone plus and performed in two steps: Firstly, predenaturation for 3 min and 45 cycles of denaturation for 3 s at 95°C, then annealing/extension for 30 s at 60°C. Gene expression was normalized by the $2^{-\Delta \Delta C_{t}}$ method, and the 16S rRNA gene was used as the normalized standard.

**Table 1:** Selected genes and primers for qRT‐PCR

Target genes ID	Discription	Primer sequence	Tm	Length (bp)
BAD_RS02255	Sugar ABC transporter substrate‐binding protein	F:AGGAAGGTGCTTTGATGGG	57.2	116
		R:GGCGTATTTCTCCTGATTTGA	57.2	
BAD_RS02260	Sugar ABC transporter permease	F:CTTCGTGCCGTATGTCGTTT	58.7	246
		R:TGGCTGCTTCATACAGTTCC	57.9	
BAD_RS00875	Phosphoenolpyruvate‐protein phosphotransferase	F:TGTTCCGTACCGAATTCCTGT	59.5	117
		R:TGCGGATCACGACCTTCTT	58.5	
BAD_RS01940	PTS beta‐glucoside transporter subunit EIIBCA	F:ACGCTCGGCTACGACTTCAT	59.9	200
		R:AGCTTGTACCGCAGGTGGAT	58.5	
BAD_RS06365	Beta‐glucosidase	F:CTTCTACATCACCGCCTACCA	57.8	167
		R:TATCGAGGACCACGTTCTTAAA	57.2	
BAD_RS02270	Beta‐xylosidase	F:CCAGCCAGCTTGATATGAGAG	57.6	154
		R:TCGGCGGTGACCAAATAA	57.8	
BAD_RS03990	Glutamate synthase [NADPH] large subunit	F:TCGTGCATTCCCGCTTC	58.0	103
		R:TTGCCTTGGATGGTGTTGA	57.4	
BAD_RS04070	Dihydroorotate oxidase	F:GAACAGCACGAATGGAAGCA	59.3	188
		R:GCAGTACGGATGCCAGGATT	59.9	
BAD_RS07900	dTDP‐glucose 4,6‐dehydratase	F:GTTCACGGAGCATACCCCATA	59.9	147
		R:TGCTGGAAGGGACCGTAGTT	59.5	
BAD_RS08125	Molecular chaperone DnaK	F:ACCGACTGGACCGTTGAGAT	59.0	156
		R:CTGGGCGTCGTTGAAGTATG	59.0	
BAD_RS07405	Multiple sugar‐binding transport system permease	F:CAACGCCTTCAAGAACACC	56.1	200
		R:GACCCACCTGTGCCTCCAT	59.9	
BAD_RS01610	Xylanase	F:ATGACGGAAAGCCGCATGT	57.6	163
		R:CGGTCGTGGGTCAGGAAGA	59.6	
BAD_RS03215	ABC transporter permease	F:CTCATCTCGCTCGTCTCCG	58.8	200
		R:GGTACTTTGACCGCTCTGC	55.4	
BAD_RS06375	MFS transporter	F:GAACATGATGATCGCACCG	57.3	190
		R:GGAAAGACCCATAGCCACA	55.4	
BAD_RS02300	Membrane‐associated protein	F:CTGCTCGCTCGTCCTCGTC	60.7	170
		R:TCTCCACTTTGCCCGTTCC	61.2	
BAD_RS03325	ABC transporter ATP‐binding protein	F:GTCCTTCGCCATCGAGCCT	63.4	158
		R:CACTGCTGCGGGGTGAAAT	63.9	
	16S rRNA	F:GAGCGAACAGGATTAGATAC	57.6	144
		R:TCTTTGAGTTTTAGCCTTGC	58.0	

### 2.7 Metabolites extraction

The sample of 100 μl was accurately removed and placed in EP tube, and 300 μl methanol was added to start extraction, add 20 μl internal standard substances and followed by vortex for 30 s. Then, the mixture tube was immersed into the ultrasonic bath with ice water and ultrasonically incubated in ice water for 10.0 min and incubated for 1 hr at −20°C to precipitate proteins. Then, the mixture was centrifuged at 11,390 *g* for 15 min at 4°C. About 200 μl of supernatant sample was transferred to a fresh 2 ml LC/MS glass vial, 20 μl from supernatant of each sample was marked as QC samples, and another supernatant was used for the UHPLC‐QTOF‐MS analysis. All experiments were carried out in triplicate.

### 2.8 LC‐MS/MS analysis

The UHPLC system (1290, Agilent Technologies) with a UPLC BEH Amide column (1.7 μm 2.1 × 100 mm, Waters) coupled with Triple TOF 5600 (Q‐TOF, AB Sciex) was used for LC‐MS/MS analyses. 25 mM NH_4_OAc and 25 mM NH_4_OH in water (pH = 9.75) (A) and acetonitrile (B) were used as the mobile phase. The elution gradient was as follows: 0 min, 95% B; 7 min, 65% B; 9 min, 40% B; 9.1 min, 95% B; and 12 min, 95% B. The flow rate of the mobile phase was 0.5 ml/min. The injection volume of analytical solution was 3 μl. The Triple‐TOF‐MS was used for its ability to acquire MS/MS spectra on an information‐dependent basis (IDA) during an LC/MS experiment. In this mode, the full scan survey MS data as it collects and triggers the acquisition of MS/MS spectra depending on preselected criteria were surveyed by the acquisition software (Analyst TF 1.7, AB Sciex; Fraga, Clowers, Moore, & Zink, 2010). In each cycle, 12 precursor ions with intensity greater than 100 were chosen for fragmentation at collision energy (CE) of 30 V (15 MS/MS events with product ion accumulation time of 50 msec each). ESI source conditions were set as following: ion source gas 1 as 60 Psi, ion source gas 2 as 60 Psi, Curtain gas as 35 Psi, source temperature 650°C, ion spray voltage floating (ISVF) 5,000 or −4,000 V in positive or negative modes, respectively.

### 2.9 Data processing and analysis

The mzXML format were obtained by using ProteoWizard to convert MS raw data files, and processed by R package XCMS (version 3.2). The processed results generated a data matrix consisted of retention time (RT), massto‐charge ratio (*m*/*z*) values, and peak intensity. R package CAMERA was used for peak annotation after XCMS data processing (Kim et al., 2013). The metabolites were identified by In‐house MS_2_ database.

## 3 RESULTS

### 3.1 Growth characteristics of *B. adolescentis* 15703

**Figure 1:**
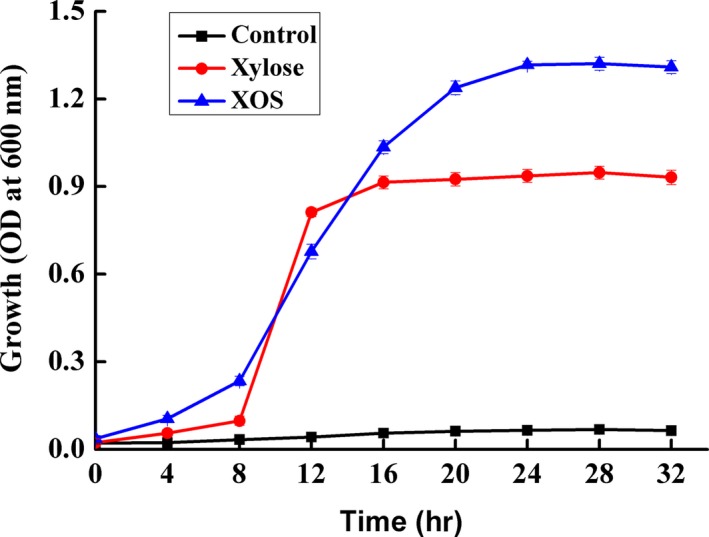
Growth of *Bifidobacterium adolescentis* 15703 on Xylo‐oligosaccharide (XOS), xylose, and control medium (no carbohydrate)

The growth characteristics of *B. adolescentis* 15703 on xylose and XOS are presented in Figure 1. *Bifidobacterium adolescentis* grew better on xylose and XOS as carbon sources compared with CK (control group) without carbon source. Also, a rapid growth rate was observed when XOS was used compared with xylose. The growth yield (stable phase) on XOS was about 1.5‐fold greater than that on xylose, indicating that XOS was more preferred by *B. adolescentis*.

### 3.2 RNA‐seq analysis and differential gene expression

**Figure 2:**
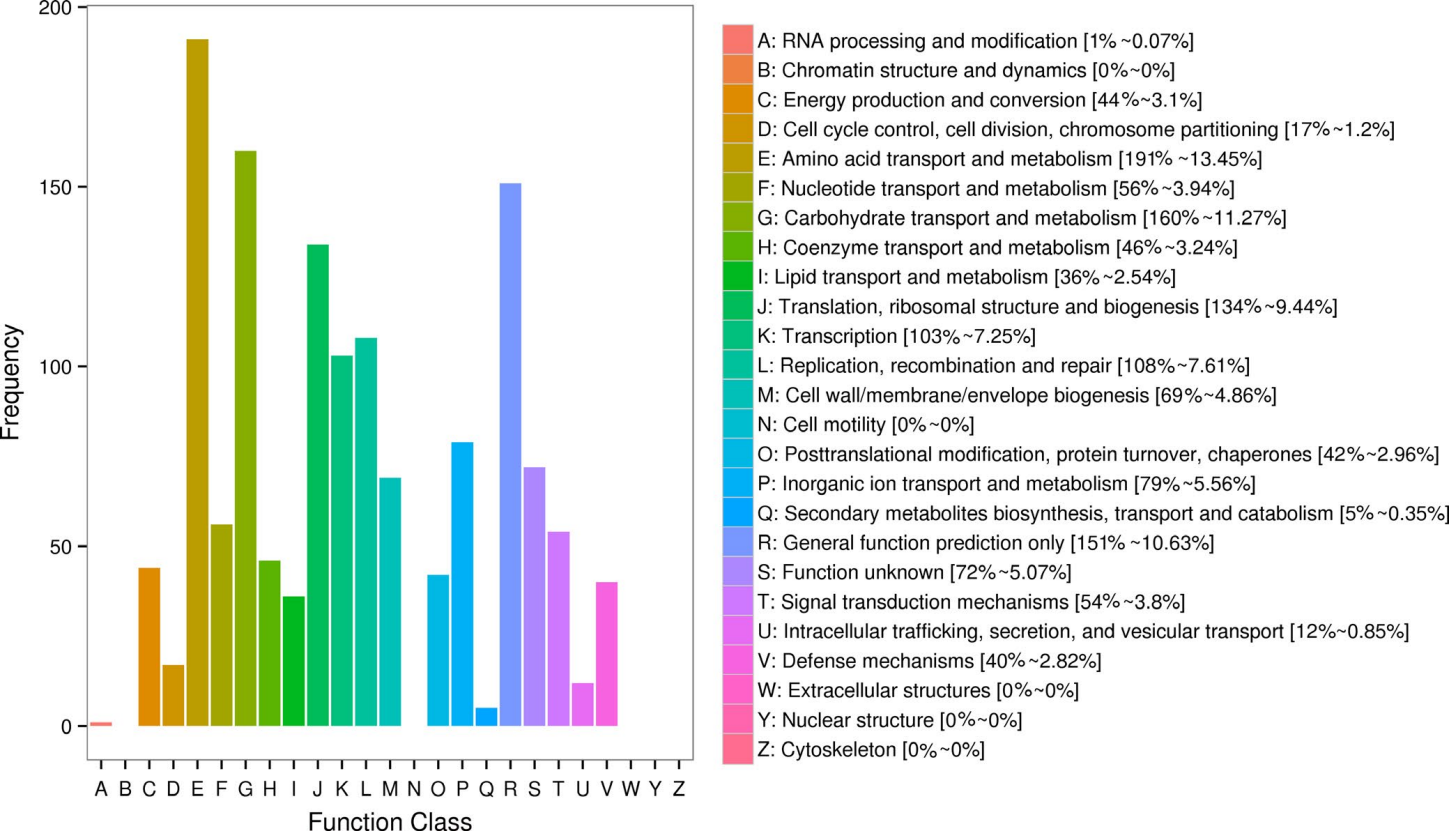
COG function classification of genes in *Bifidobacterium adolescentis* 15703. The categories of the COG are shown on the horizontal axis, and gene numbers are plotted on the vertical axis

From the RNA‐seq analysis data, it can be seen that over 99% of the reads were aligned to encoding regions of the *B. adolescentis*. Genes were assigned to 25 functional groups, which were annotated in COG database (Figure 2). Among these classifications, the largest group was amino acid transport and metabolism (191, 13.45%), followed by carbohydrate transport and metabolism (160, 11.27%) and general function prediction (151, 10.63%).

**Figure 3:**
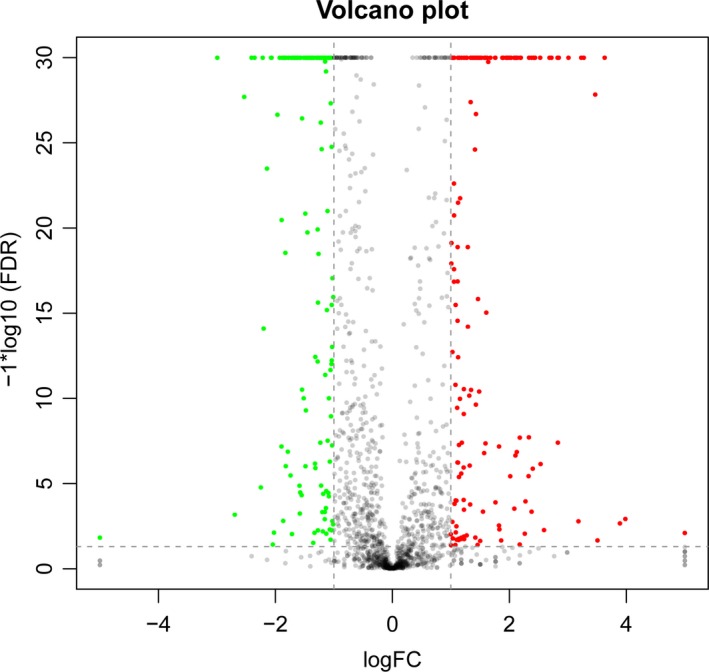
Change level of global DEGs between XOS and xylose treatment. Red dot: upregulated; green dot: downregulated; black dot: not DEGs

A total number of 302 DEGs were identified for *B. adolescentis* grown on xylose and XOS, including 158 upregulated genes and 144 downregulated genes (Figure 3). The top 10 upregulated genes and 10 downregulated genes of xylose and XOS treatments are presented in Table 2. Four genes of the top 10 upregulated genes encode ABC and MFS transporters. Among the remaining genes, two genes encode hsp20/alpha crystallin family protein and ATP‐dependent chaperone ClpB, two genes encode RNA polymerase sigma factor and death‐on‐curing protein, other two genes encode enzyme proteins belonging to multiple sugar‐binding transport system permease and shikimate kinase. Five genes of the top 10 downregulated genes encode structure protein, including penicillin‐binding protein, von willebrand factor type A domain protein, fhiA protein, arginine repressor DUF4956, domain‐containing protein, three genes are associated with membrane transport, including peptide ABC transporter ATP‐binding protein, ABC transporter permease, and membrane spanning polysaccharide biosynthesis protein, while two genes encode O‐antigen polymerase and hypothetical protein.

**Table 2:** Top 10 significantly upregulated and downregulated genes during growth of *Bifidobacterium adolescentis* 15703 on XOS compared with xylose assessed by RNAseq

Gene no.^a^	Log_2_ (Fc)^b^	Annotation^c^	Linear FMPK value^d^	
			XOS	Xylose
BAD_RS07405	3.63↑	Multiple sugar‐binding transport system permease	160.73	12.98
BAD_RS05940	3.51↑	MFS transporter	6.71	0.59
BAD_RS01050	3.47↑	Shikimate kinase	173.6	15.68
BAD_RS07410	3.27↑	ABC transporter permease	159.79	16.53
BAD_RS07415	3.23↑	ABC transporter, solute‐binding protein	486.74	51.88
BAD_RS08735	3.18↑	RNA polymerase sigma factor	18.24	2.01
BAD_RS00260	3.01↑	hsp20/alpha crystallin family protein	13,180.79	1,634.87
BAD_RS02255	2.85↑	Sugar ABC transporter substrate‐binding protein	7,109.23	989.28
BAD_RS05015	2.83↑	Death‐on‐curing protein	89.91	12.64
BAD_RS07715	2.83↑	ATP‐dependent chaperone ClpB	3,432.39	484.32
BAD_RS00210	2.08↓	Penicillin‐binding protein	120.14	505.44
BAD_RS07300	2.14↓	O‐antigen polymerase	28.92	127.77
BAD_RS02985	2.20↓	Hypothetical protein	32.4	148.91
BAD_RS02975	2.22↓	von Willebrand factor type A domain protein	44.03	204.58
BAD_RS08925	2.25↓	fhiA protein	22.97	109.11
BAD_RS03210	2.35↓	Peptide ABC transporter ATP‐binding protein	97.09	496.58
BAD_RS07325	2.41↓	Membrane spanning polysaccharide biosynthesis protein	22.11	117.21
BAD_RS04925	2.53↓	Arginine repressor	43.27	250.28
BAD_RS02140	2.69↓	DUF4956 domain‐containing protein	3.07	19.86
BAD_RS03215	2.99↓	ABC transporter permease	60.43	480.91

a Gene number referenced as *B. adolescentis* 15703 being alphabet and a five‐digit number.

b Significance of fold change data is judged by having a *p* value of no more than .01.

c Gene annotations were blasted against Swiss prot.

d FPKM (fragments per kilobase of exon per million fragments mapped) values for cultures on media with Xylose or XOS.

### 3.3 KEGG pathway mapping of DEGs

**Figure 4:**
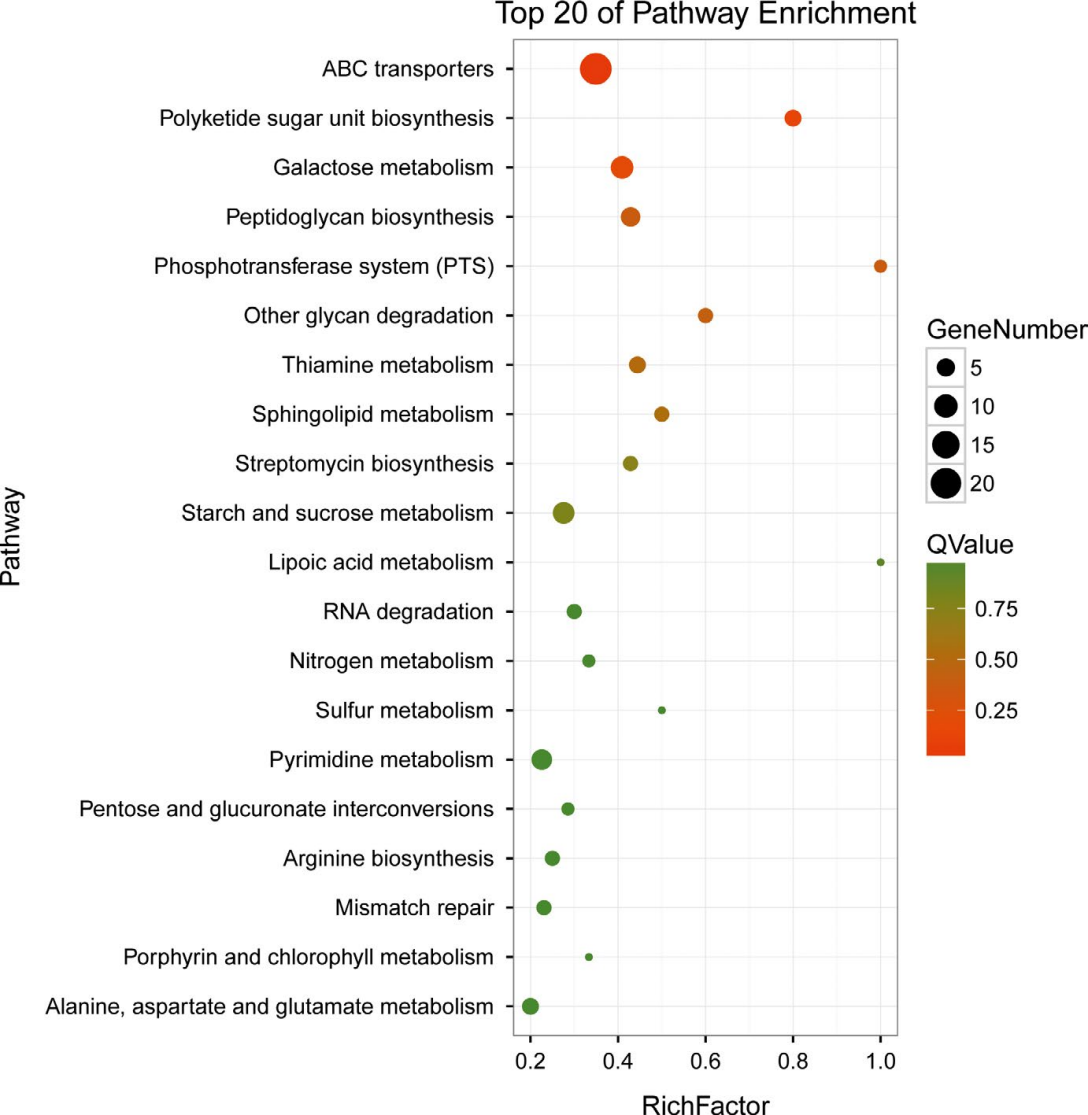
KEGG pathway enrichment analysis of DEGs (XOS vs. xylose). The vertical axis indicates the name of KEGG pathway, and the horizontal axis indicates the Rich factor. The dot size indicates the number of differentially expressed genes in the pathway, and the color of the dots corresponds to different *Q* values

The DEGs involved in biological functions were further analyzed by KEGG pathways, and 20 pathways were predicted (Figure 4). ABC transporters, galactose metabolism, peptidoglycan biosynthesis pyrimidine metabolism, starch, and sucrose metabolism are the highly represented categories.

The DEGs involved in the ABC transporters are shown in Table 3. In the ABC transporter pathway (ko02010), 27 genes were significantly upregulated. Genes 07405, 07410, 02260, 08210, 08205, 00815, 00810, 08280, 03705, 08275, 06685, 08210 encoded ABC transporter permease, Genes 07415, 02255, 01495, 00390, 00805, 08285, 00990, 06680 encoded ABC transporter substrate‐binding protein, Gene 02265, 04090, 00495, 03325, 08375 encoded ABC transporter ATP‐binding protein, 07050 and 00340 encoded ABC transporter, while nine genes (02355, 02470 and 03935), which are ABC transporter‐related genes, significantly downregulated after XOS treatment.

**Table 3:** DEGs involved in related ABC transporter during growth of *Bifidobacterium adolescentis* 15703 on XOS compared with xylose assessed by RNAseq

Gene no.	Log_2_ (Fc)	Symbol	Annotation	Linear FMPK value	
				XOS	Xylose
BAD_RS07405	3.02↑	amyC	Multiple sugar‐binding transport system permease	105.08	12.98
BAD_RS07410	3.27↑	amyD	ABC transporter permease	159.79	15.53
BAD_RS07415	2.80↑	mdxE	ABC transporter, solute‐binding protein	360.4	51.88
BAD_RS02255	3.00↑	yurO	Sugar ABC transporter substrate‐binding protein	7,940.17	989.28
BAD_RS01495	2.69↑	TP_0034	ABC transporter substrate‐binding protein	2,060.19	319.83
BAD_RS00390	2.33↑	BR1785	Branched‐chain amino acid ABC transporter substrate‐binding protein	30.52	6.05
BAD_RS02265	2.38↑	yurM	Thiamine ABC transporter ATP‐binding protein	2,679.25	514.59
BAD_RS02260	2.34↑	malF	Sugar ABC transporter permease	2,356.22	466
BAD_RS00385	2.28↑	livF	ABC‐type branched‐chain amino acid transport systems ATPase component	25.2	5.2
BAD_RS08210	1.99↑	amyD	Permease of ABC transporter possibly for oligosaccharides	3,704.78	933.34
BAD_RS00805	1.95↑	yurO	Solute‐binding protein of ABC transporter system	2,384.03	618.73
BAD_RS08205	1.88↑	amyC	Sugar ABC transporter permease	2,223.03	603.5
BAD_RS00495	1.76↑	MT1311	Multidrug ABC transporter ATP‐binding protein	276.15	81.69
BAD_RS07050	1.58↑	lipO	ABC transporter	10,020.43	3,361.71
BAD_RS08285	1.52↑	ugpB	ABC transporter, solute‐binding protein	1,971.94	686.94
BAD_RS00815	1.48↑	araQ	Sugar ABC transporter permease	911.12	326.41
BAD_RS08280	1.46↑	msmF	Sugar ABC transporter permease	1,353.65	491.14
BAD_RS00810	1.45↑	yurN	Sugar ABC transporter permease	762.04	278.86
BAD_RS03705	1.35↑	—	ABC transporter permease	91.4	35.95
BAD_RS00990	1.33↑	—	ABC transporter substrate‐binding protein	34.99	13.91
BAD_RS03325	1.29↑	MJ1508	ABC transporter ATP‐binding protein	209.24	85.46
BAD_RS08275	1.27↑	amyC	ABC transporter permease	1,000.47	413.73
BAD_RS04090	1.14↑	TM_0352	Macrolide ABC transporter ATP‐binding protein	33.93	15.42
BAD_RS00340	1.05↑	Pip	ABC transporter	228.58	109.76
BAD_RS06680	1.27↑	yxeM	Amino acid ABC transporter substrate‐binding protein	552.97	228.62
BAD_RS06685	1.05↑	tcyL	ABC transporter permease	409.27	196.97
BAD_RS00370	1.04↑	livH	Branched‐chain amino acid ABC‐type transport system permease components	17.92	8.75
BAD_RS08375	1.02↑	msmX	ABC transporter ATP‐binding protein	38,386.82	18,870.88
BAD_RS03070	1.01↓	artQ	Glutamine ABC transporter permease	89.94	181.07
BAD_RS02355	1.03↓	braC	Solute‐binding protein of ABC transporter for branched‐chain amino acids	83.57	171.31
BAD_RS02470	1.05↓	ftsX	ABC transporter permease	240.08	497.88
BAD_RS03935	1.07↓	rbsA1	ABC transporter ATP‐binding protein	10.55	22.14
BAD_RS04785	1.22↓	yclH	ATP‐binding protein of ABC transporter similar to Vex2	40.66	94.55
BAD_RS05605	1.28↓	—	Sugar ABC transporter substrate‐binding protein	101.96	247.02
BAD_RS03930	1.33↓	—	Cobalt ABC transporter permease	12.88	32.39
BAD_RS03210	2.35↓	lolD	Peptide ABC transporter ATP‐binding protein	97.09	496.58
BAD_RS03215	2.76↓	macB	ABC transporter permease	71.17	480.91

The DEGs involved in carbohydrate metabolism are shown in Table 4. Compared with xylose treatment, five genes (08325, 07400, 07395, 06400, 08455) encoded beta‐galactosidase and two genes (08195, 08270) encoded alpha‐amylase related to galactose metabolism pathway (ko00052) significantly upregulated after XOS treatment. Also, three genes (02270, 08480, 02400) expressed key enzymes (beta‐xylosidase, beta‐glucosidase) involved in starch and sucrose metabolism (ko00500) significantly upregulated after XOS treatment. Gene 05480 coded mannan endo‐1,4‐beta‐mannosidase involved in fructose and mannose metabolism (ko00051). 01050 coded shikimate kinase, 01040 coded 6‐phosphogluconate dehydrogenase, 02150 coded lactaldehyde reductase, 07445 coded L‐ribulose‐5‐phosphate 4‐epimerase, 01580 coded UDP‐N‐acetylenolpyruvoylglucosamine reductase, which involved in biosynthesis of antibiotics (ko01130), microbial metabolism in diverse environments (ko01120) carbon metabolism (ko01200), pentose phosphate pathway (ko00030), glyoxylate and dicarboxylate metabolism (ko00630), propanoate metabolism (ko00640), and pentose and glucuronate interconversions (ko00040).

**Table 4:** DEGs involved in related carbohydrate metabolism in KEGG pathway during growth of *Bifidobacterium adolescentis* 15703 on XOS compared with xylose assessed by RNAseq

Gene no.	Log_2_ (Fc)	Symbol	Annotation	Linear FMPK value		KEGG pathway
				XOS	Xylose	
BAD_RS01050	3.47↑	Idnk	Shikimate kinase	173.6	15.68	ko01100
BAD_RS07400	2.44↑	BGAL16	Beta‐galactosidase	70.88	13.06	ko01100/ko00052/ko00600/ko00511
BAD_RS01040	2.42↑	gnd	6‐phosphogluconate dehydrogenase	240.52	45.07	ko01100/ko01110/ko01130/ko01120//ko01200/ko00030/ko00480
BAD_RS08195	2.20↑	malL	Alpha‐amylase	3,359.55	733.05	ko01100/ko00500/ko00052
BAD_RS02270	2.08↑	xynB	Beta‐xylosidase	1,066.15	251.3	ko01100/ko00500/ko00052
BAD_RS08325	1.97↑	LacZ	Beta‐galactosidase	98.72	25.2	ko01100/ko00052/ko00600/ko00511
BAD_RS02150	1.68↑	fucO	Lactaldehyde reductase	5,736.74	1,795.21	ko01120/ko00630/ko00640
BAD_RS08455	1.38↑	lacZ	Beta‐galactosidase	329.16	126.46	ko01100/ko00052/ko00600/ko00511
BAD_RS08270	1.34↑	malL	Alpha‐amylase	1,248.09	493.04	ko01100/ko00500/ko00052
BAD_RS07445	1.26↑	ulaF	L‐ribulose‐5‐phosphate 4‐epimerase	549.87	229.09	ko01100/ko00040
BAD_RS06365	1.23↑	exgA	Beta‐glucosidase	1,112.17	475.02	ko00500
BAD_RS06400	1.19↑	bgaB	Beta‐galactosidase	45.11	19.79	ko00052
BAD_RS01695	1.18↑	—	Sulfurtransferase	20.92	9.24	ko01100/ko00040
BAD_RS02400	1.16↑	malL	Alpha‐glucosidase	76.25	34.21	ko01100/ko00500/ko00052
BAD_RS06090	1.14↑	cscA	Beta‐(1‐2)‐fructofuranosidase	47.64	21.63	ko01100/ko00500/ko00052
BAD_RS05480	1.11↑	BAD_1030	Mannan endo‐1,4‐beta‐mannosidase	29.85	13.79	ko00051
BAD_RS01580	1.08↑	murB	UDP‐N‐acetylenolpyruvoylglucosamine reductase	198.47	93.71	ko01100/ko00520/ko00550
BAD_RS08480	1.06↑	bglB	Beta‐glucosidase	137.7	66.25	ko01100/ko01110/ko00500/ko00460
BAD_RS07395	1.05↑	bgaB	Beta‐galactosidase I	175.7	84.64	ko00052
BAD_RS05595	1.05↓	acn	Aconitate hydratase	137.1	283.08	ko01100/ko01110/ko01130/ko01230/ko01120/ko01200/ko01210/ko00630/ko00020
BAD_RS07575	1.25↓	glgE	Alpha‐1,4‐glucan‐maltose‐1‐phosphate maltosyltransferase	461.18	1,093.58	ko01100/ko00500

### 3.4 Validation of transcript abundance using qRT‐PCR

**Figure 5:**
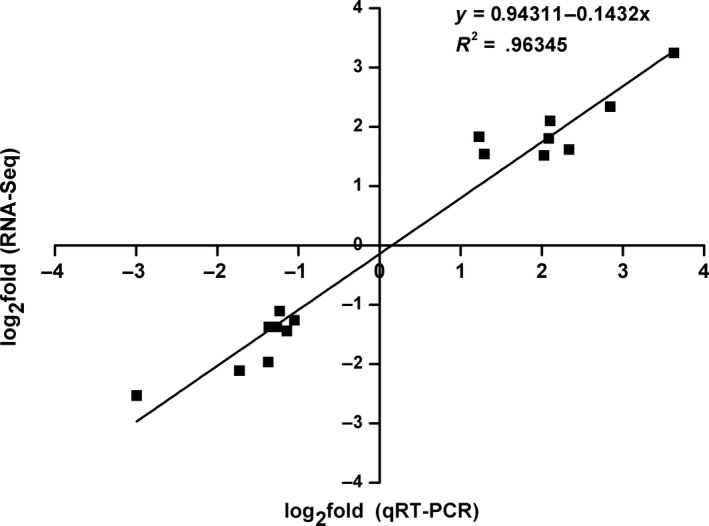
Quantitative real‐time PCR (qRT‐PCR) validations of DEGs against RNA‐seq

To verify the RNA‐Seq results, the mRNA expression of 16 selected candidate genes (eight upregulated and eight downregulated) was measured by qRT‐PCR. The expression levels of 16 DEGs with qRT‐PCR were compared with those of DEGs with RNA‐Seq by the linear fitting. A significant correlation (*R* ^2^ = 0.96345) was found between the RNA‐Seq and qRT‐PCR (Figure 5). The qRT‐PCR results are consistent with their transcript abundance in RNA‐seq, which verified the accuracy of the DEGs from RNA‐seq analyses.

### 3.5 Metabolite profile

The metabolites profiling of *B. adolescentis* 15703 was performed using LC‐MS. The primary metabolites are amino acids, organic acids, fatty acid, polyhydroxy acids, sugars, phosphates, polyols, and *N*‐compounds. A total number of 157 different metabolites (MS_2_) were identified (*p* < .05, log_2_FC >1) for xylose and XOS treatments, including 79 upregulated metabolites and 78 downregulated metabolites. The top 10 upregulated and 10 downregulated metabolites for xylose and XOS treatments are presented in Table 5. Six metabolites of the top 10 upregulated metabolites are acids, including linolenic acid, epoxy stearic acid, myristic acid, uric acid, palmitoleic acid, and oleic acid. The remaining metabolites are D‐sorbitol 6‐phosphate, 3‐prenyl‐4‐Hydroxyacetophenone, L‐threonine, and L‐phenylalanyl‐L‐proline. Three metabolites of the top 10 downregulated metabolites are 3‐Hydroxymandelic acid, 3‐Dehydroshikimic acid, vanillylmandelic acid, and other remaining metabolites are flutamide, dihydroxyfumarate, hydroxyhydroquinone, quinone, 3′‐O‐Methylinosine, N‐acetyl‐L‐alanine, and norepinephrine.

**Table 5:** Top 10 significantly upregulated and downregulated metabolites during growth of *Bifidobacterium adolescentis* 15703 on XOS compared with xylose assessed by metabolome

Meta ID	log_2_FC	MS_2_ name	mzmed	rtmed
meta_736	9.127↑	All cis‐(6, 9, 12)‐Linolenic acid	277.222	45.101
meta_761	5.107↑	D‐Sorbitol 6‐phosphate	283.128	44.744
meta_428	3.978↑	3‐Prenyl‐4‐Hydroxyacetophenone	220.130	250.897
meta_827	3.251↑	Nname, cis‐9, 10‐Epoxystearic acid	297.248	62.159
meta_468	3.091↑	Myristic acid	227.205	45.097
meta_458	2.915↑	Uric acid	227.036	88.723
meta_58	2.848↑	L‐Threonine	118.053	239.084
meta_607	2.675↑	cis‐9‐Palmitoleic acid	253.221	44.427
meta_741	2.566↑	L‐phenylalanyl‐L‐proline	278.144	114.569
meta_753	2.533↑	Oleic acid	281.253	43.288
meta_721	3.087↓	Flutamide	275.064	118.82
meta_459	3.105↓	3‐Hydroxymandelic acid	227.061	47.247
meta_192	3.107↓	Dihydroxyfumarate	169.043	48.585
meta_65	3.131↓	Hydroxyhydroquinone	125.027	206.664
meta_182	3.220↓	Quinone	167.039	48.545
meta_750	3.387↓	3′‐O‐Methylinosine	281.088	26.536
meta_1000	4.285↓	3‐Dehydroshikimic acid	343.068	166.570
meta_622	4.393↓	Vanillylmandelic acid	257.071	192.365
meta_289	4.855↓	N‐Acetyl‐L‐alanine	190.075	104.461
meta_473	6.446↓	Norepinephrine	228.092	67.598

### 3.6 KEGG pathway mapping of metabolites

**Figure 6:**
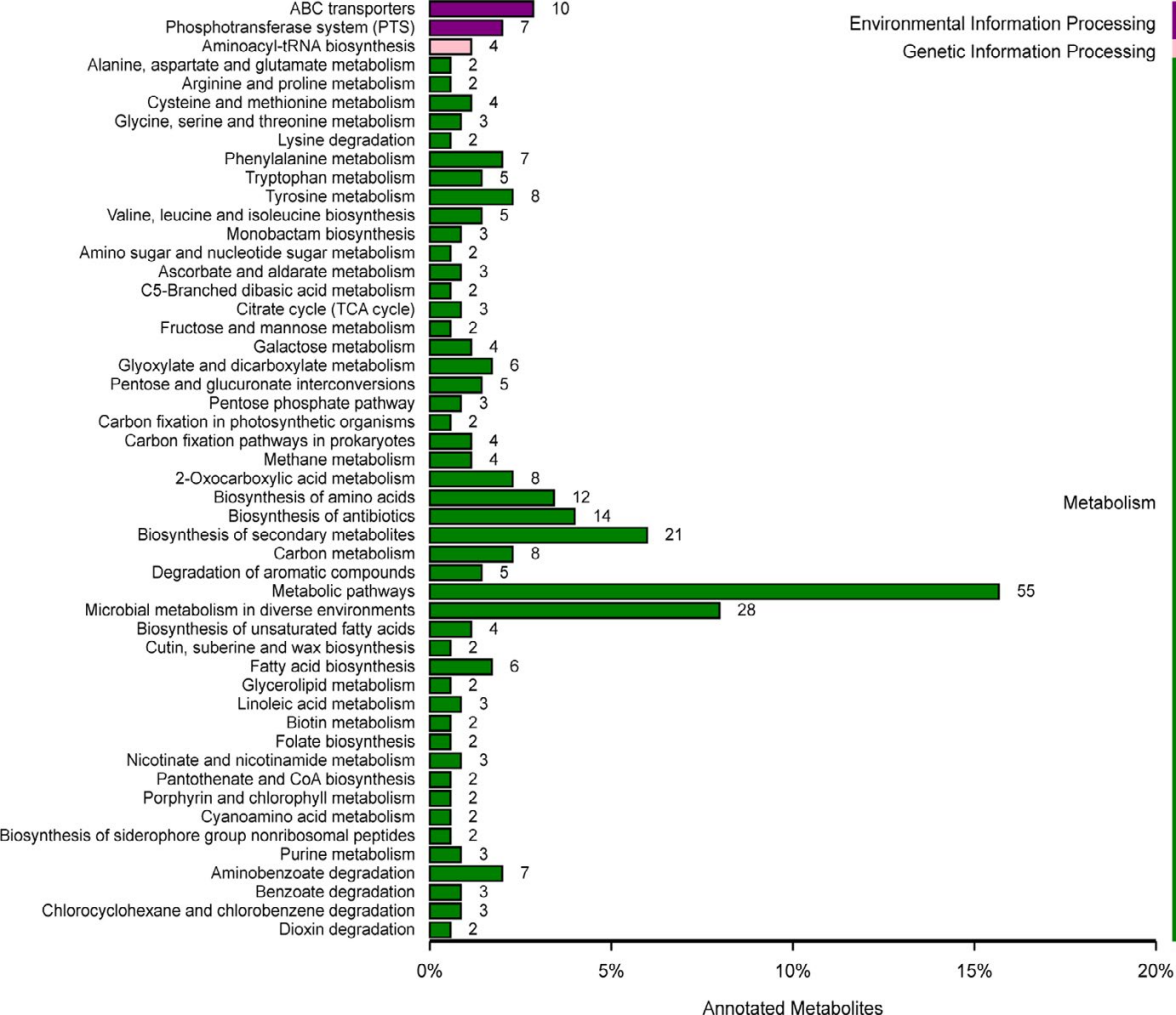
The annotated and classified results of the differential metabolite in KEGG pathway

A total number of 50 enriched KEGG pathways were predicted, which were associated with different metabolites (Figure 6). The 50 pathways were classified as environmental information processing, genetic information processing, and metabolism. The environmental information processing included ABC transporters and phosphotransferase system. In metabolism processing, microbial metabolism in diverse environments and biosynthesis of unsaturated fatty acids are the most highly represented (Figure 6).

Different metabolites involved in carbohydrate transport and metabolism are shown in Table 6. Compared with xylose treatment, 10 metabolites (58, 911, 166, 1695, 82, 651, 15, 81, 348, 376), which are L‐threonine, cellobiose, D‐Mannose, maltotriose, L‐isoleucine, D‐biotin, glycerol, L‐leucine, N‐acetyl‐D‐glucosamine, D‐ribose are significantly different in the ABC transporters pathway (ko02010) for XOS treatment. Also, five metabolites, including D‐sorbitol 6‐phosphate, cellobiose, D‐mannose, L‐ascorbic acid, and N‐acetyl‐D‐glucosamine are significantly different in the phosphotransferase system (PTS; ko02060) for XOS treatment compared with xylose treatment. Isocitrate, citrate, and pyruvate are significantly different in Citrate cycle (TCA; ko00020). Sedoheptulose, isocitrate, tetrahydrofolate are significantly different in carbon fixation pathways in prokaryotes (ko00720). The remaining metabolites, which are galactinol, L‐ribulose, D‐glucose 6‐phosphate, 2‐keto‐D‐gluconic acid, and L‐threonine involved in galactose metabolism (ko00052), starch and sucrose metabolism (ko00500), pentose phosphate pathway (ko00030), biosynthesis of amino acids (ko01230), biosynthesis of secondary metabolites (ko01110), and so on.

**Table 6:** Metabolites involved in related carbohydrate transport and metabolism in KEGG pathway during growth of *Bifidobacterium adolescentis* 15703 on XOS compared with xylose assessed by metabolome

ID	MS_2_ name	mzmed	rtmed	log_2_Fc	KEGG_pathway_annotation
meta_761	D‐Sorbitol 6‐phosphate	283.128	44.744	5.11↑	ko02060/ko00051
meta_58	L‐Threonine	118.053	239.084	2.85↑	ko00260/ko01130/ko01230/ko01110/ko02010/ko00860/ko00290/ko00970/ko01100/ko01120/ko00261
meta_85	D‐Xylulose	131.038	357.043	2.45↑	ko00040/ko01100
meta_2004	Galactinol	683.235	370.331	2.26↑	ko00052
meta_991	Cellobiose	341.113	281.354	2.14↑	ko02010/ko00500/ko02060/ko01100
meta_227	2‐keto‐D‐Gluconic acid	175.028	242.935	2.08↑	ko00030/ko01100/ko01120
meta_135	Ribitol	151.064	232.243	1.91↑	ko00740/ko00040/ko01100
meta_166	D‐Mannose	161.048	418.573	1.41↑	ko00520/ko02060/ko02010/ko01100/ko00052/ko00051
meta_211	Isocitrate	173.012	478.821	1.41↑	ko01210/ko00720/ko01200/ko01100/ko01120/ko00020/ko00630/ko01230/ko01130/ko01110
meta_2096	Stachyose	725.246	464.357	1.36↑	ko00052
meta_1317	Tetrahydrofolate	444.157	237.902	1.35↑	ko00720/ko01100/ko01200/ko01120/ko00970/ko00670/ko00790/ko00680/ko00260/
meta_1695	Maltotriose	563.190	430.279	1.29↑	ko02010
meta_295	Sedoheptulose	191.060	73.835	1.26↑	ko00710
meta_82	L‐Isoleucine	130.090	221.442	1.15↑	ko01110/ko00460/ko01130/ko01230/ko00280/ko02010/ko01100/ko00290/ko00970/ko01210
meta_651	D‐Biotin	260.109	104.804	1.13↑	ko02010/ko01100/ko00780
meta_15	Glycerol	91.042	107.553	1.05↑	ko00040/ko02010/ko01100/ko00561/ko00052
meta_84	L‐Ribulose	131.037	372.505	1.02↑	ko00040/ko01100
meta_312	L‐Ascorbic acid	197.006	45.633	1.05↓	ko01120/ko01100/ko01110/ko00053/ko00480/ko02060
meta_81	L‐Leucine	130.090	180.456	1.08↓	ko01110/ko01230/ko00280/ko02010/ko01100/ko00970/ko00290/ko01210/
meta_1	Dihydroxyacetone	71.016	198.357	1.21↓	ko00561/ko01200/ko01100/ko01120/ko00680
meta_533	D‐Glucose 6‐phosphate	241.007	91.875	1.24↓	ko00500/ko02060/ko05111/ko01130/ko02020/ko00521/ko00524/ko00562/ko01100
meta_74	Citraconic acid	129.022	73.135	1.29↓	ko00630/ko00660/ko01210/ko01200/ko01100/ko00290
meta_4	Glycolate	75.010	262.283	1.33↓	ko00361/ko00625/ko00630/ko01130/ko01110/ko01120/ko01200/ko01100
meta_348	N‐Acetyl‐D‐glucosamine	202.076	65.036	1.42↓	ko02010/ko02060/ko00520/ko01100
meta_293	Citrate	191.022	376.386	2.50↓	ko00020/ko00630/ko01230/ko00250/ko01130/ko01210/ko02020/ko00720/ko01200/ko01100/ko01120
meta_376	D‐Ribose	209.070	204.675	2.61↓	ko00030/ko02030/ko02010
meta_8	Pyruvate	87.011	54.515	2.74↓	ko01220/ko01110/ko00260/ko01130/ko00010/ko00770/ko00620/ko01100/ko01200/ko00730/ko01502/ko00622/ko00660/ko00270/ko00760/ko00710/ko00250/ko00020/ko00440/ko00040/ko01210/ko00430/ko00030)/ko01230/ko00900/ko00680/ko00650/ko00630/ko01120/ko00362/ko00360/ko00621/ko00290/ko00350/ko00053/ko00473/ko02060/ko00330/ko00261/ko00720

## 4 DISCUSSION

### 4.1 *B. adolescentis* responses to xylose and XOS

To investigate the growth performance of *B. adolescentis* on xylose and XOS as carbon sources, growth curves of strain were determined. *Bifidobacterium adolescentis* showed a strong capacity in utilizing of XOS to proliferate, which may indicate that most genes and metabolites in *B. adolescentis* are related to XOS transport and metabolism. XOS needs to be degraded into xylose before it can be metabolized (Broekaert et al., 2011). Therefore, degradation of XOS is complicated, resulting in a relatively longer lag phase when used as a substrate compared with xylose.

### 4.2 Gene prosperities of *B. adolescentis* ATCC 15703

*Bifidobacterium adolescentis* ATCC 15703, the predominant species of *Bifidobacterium*, was isolated from the human GIT (Pokusaeva et al., 2011). Currently, more than 40 bifidobacterial genomes including those of *B. adolescentis* 15703 strain have been completely sequenced and annotated in the NCBI database (Sayers et al., 2019; Schell et al., 2002). The complete genome size of the current *B. adolescentis* ATCC 15703 is 2,089,645 bp, with gene number 1701, protein 1631, and G‐C content of 59% (Bondue & Delcenserie, 2015).

A recent study performed on the genome sequences from 47 *Bifidobacterium* species found that 5.5% of the core bifidobacterial genomic coding sequences were associated with carbohydrate metabolism (Milani et al., 2015). The bifidobacterial genome encode a variety of carbohydrate‐modifying enzymes, such as glycosyl hydrolases, sugar ABC transporters, and PEP‐PTS system components, all of which are required for the metabolism of carbohydrates (Chen et al., 2019; Liu et al., 2014). Majority of these genes are devoted to carbohydrate uptake, by means of ABC transporters and permeases (Table 3). According to the KEGG and COG classifications, most of genes in *B. adolescentis* 15703 are associated with carbohydrate metabolism and could imply relative importance of carbohydrate utilization.

### 4.3 Comparison of transport pathways of *B. adolescentis* grown on xylose and XOS

Bifidobacteria internalize carbohydrates by ATP‐dependent ABC transporters and PEP‐PTS systems (Degnan & Macfarlane, 1993; Turroni et al., 2012). However, a minority of sugars utilized by bifidobacteria are believed to be internalized via a PEP‐PTS (Degnan & Macfarlane, 1993; Maze, O'Connell‐Motherway, Fitzgerald, Deutscher, & Sinderen, 2007). Compared with xylose treatment, PTS beta‐glucoside transporter subunit EIIBCA (encoded by BAD_RS01940) and phosphoenolpyruvate‐protein phosphotransferase (encoded by BAD_RS00875) were downregulated in *B. adolescentis* 15703 grown on XOS. Meanwhile, metabolites including upregulated D‐Sorbitol 6‐phosphate (meta_761), D‐mannose (meta_166) and downregulated L‐ascorbic acid (meta_312), D‐Glucose 6‐phosphate (meta_533), N‐acetyl‐D‐glucosamine (meta_348), pyruvate (meta_8) were involved in PTS system (Tables 4 and 6). Related downregulated genes and metabolites are more than upregulated ones. Therefore, uptake of the most complex sugars is possibly facilitated by specific ABC transporters.

ABC transporters couple ATP hydrolysis to efficient internalization of sugars and appear to represent the primary carbohydrate transport systems for bifidobacteria. Compared to xylose treatment, genes including the sugar transporter permease protein (encoded by BAD_RS00815, BAD_RS08280, BAD_RS00810, BAD_RS08205, BAD_RS03705, BAD_RS02260, BAD_RS07410) and transporter ATP‐binding protein (encoded by BAD_RS02265, BAD_RS00495, BAD_RS04090, BAD_RS08375) were upregulated (Table 3), the same situation occurs in metabolites, including L‐threonine (meta_58), cellobiose (meta_991), D‐mannose (meta_166), L‐isoleucine (meta_82), maltotriose (meta_1695), D‐biotin (meta_651), and glycerol (meta_15) involved in ABC transporters pathway (ko02010). Thus, XOS may enhance the sugar transport process by ABC transporters system.

### 4.4 Comparison of carbohydrate metabolism pathways of *B. adolescentis* grown on xylose and XOS

After internalization, carbohydrates can then be hydrolyzed, phosphorylated, deacetylated, and/or transglycosylated by dedicated intracellular enzymes. Glycosyl hydrolases appear to be the most critical group of enzymes for bifidobacteria. β‐glucosidases (EC3.2.1.21) are pivotal enzymes for the metabolism and homeostasis of *Bifidobacterium* because they hydrolyze small and soluble saccharides (Kelly et al., 2016; Maria, Margarita, IIlia, & Iskra, 2014). Compared with xylose, XOS upregulated genes involved in KEGG pathway (ko00052), including beta‐galactosidase (encoded BAD_RS07400, BAD_RS08325, BAD_RS08455, BAD_RS06400, BAD_RS07395). XOS was hydrolyzed by xylosidase to produce xylose, which was furtherly characterized to 5‐P‐xylulose with the action of xylose isomerase and xylulose kinase. The beta‐xylosidase (encoded BAD_RS02270) and alpha‐amylase (encoded BAD_RS08195) involved in KEGG pathway (ko00500 and ko00052) were upregulated. The upregulated genes were associated with some metabolites, including xylulose kinase, xylosidase, xylose isomerase, xylose proton symporter, which may pertain to the efficient utilization of XOS by *B. adolescentis*.

Carbohydrates were ultimately transformed to phosphoenolpyruvate through glycolysis and pentose conversions during the fermentation by *Bifidobacterium* and furtherly involved in the TCA cycle (Louis, Hold, & Flint, 2014). L‐ribulose‐5‐phosphate 4‐epimerase involved in 5‐P‐xylulose production of pentose and glucuronate interconversion pathways (ko00040) were significantly upregulated in *B. adolescentis* 15703 grown on XOS compared with that grown on xylose. However, critical DEGs related to pyruvate metabolism and the TCA cycle (ko00620; ko00020) was only aconitate hydratase (encoded by BAD_RS05595), metabolites isocitrate (meta_211) was also upregulated in *B. adolescentis* 15703 grown on XOS. Meanwhile, phosphoenolpyruvate‐protein phosphotransferase (BAD_RS00875), Citrate (meta_293) and Pyruvate (meta_8) were significantly downregulated in *B. adolescentis* 15703 grown on XOS. A lower expression of pyruvate carboxylase may lead to the reduction in oxaloacetate, which could be fermented to produce propanoic acid through the succinate pathway and is beneficial to generate lactate (Mathew, Aronsson, Karlsson, & Adlercreutz, 2018). These results indicated that XOS was preferable to be proliferated by *B. adolescentis* than xylose.

## 5 CONCLUSION

To gain insights into the regulatory networks related to XOS metabolism *B. adolescentis*, a combination of transcriptome and metabolome analyses was applied to understand the utilization and metabolism of XOS in *B. adolescentis* 15703 as well as identifying the key regulatory‐related genes and metabolites. Compared with xylose, XOS highly promoted the growth of *B. adolescentis* 15703 and the fermentation performance. XOS could enhance genes involved in transport and metabolism of carbohydrate compared with xylose. Also, the metabolomic analyses, particularly those related to metabolic biomarkers of fatty acids, amino acids, and sugars showed a similar trend of results and approved the advantages of XOS as a growth medium for *B. adolescentis* 15703 compared with xylose. Abundance of specific genes and metabolites highlighted the complex regulatory mechanisms involved in *B. adolescentis* 15703 in the presence of the XOS.

## CONFLICT OF INTEREST

The authors declare that they do not have any conflict of interest.

## ETHICAL APPROVAL

This study does not involve any human or animal testing.
